# Endoplasmic reticulum stress-related features predict the prognosis of osteosarcoma and reveal STC2 as a novel risk indicator for disease progression

**DOI:** 10.3389/fonc.2024.1453173

**Published:** 2024-07-25

**Authors:** Yongle Yu, Jiadong Yu, Zhenyu Pan

**Affiliations:** Department of Orthopedics Trauma and Microsurgery, Zhongnan Hospital of Wuhan University, Wuhan, China

**Keywords:** endoplasmic reticulum (ER) stress, ER stress risk model, osteosarcoma, immunosuppression, overall survival, tumor microenvironment

## Abstract

Endoplasmic reticulum (ER) stress exerts significant effects on cell growth, proliferation, migration, invasion, chemoresistance, and angiogenesis in various cancers. However, the impact of ER stress on the outcomes of osteosarcoma patients remains unclear. In this study, we established an ER stress risk model based on The Cancer Genome Atlas (TARGET) osteosarcoma dataset to reflect immune features and predict the prognosis of osteosarcoma patients. Survival analysis revealed significant differences in overall survival among osteosarcoma patients with different ER stress-related risk scores. Furthermore, ER stress-related risk features were significantly associated with the clinical pathological characteristics of osteosarcoma patients and could serve as independent prognostic indicators. Functional enrichment analysis indicated associations of the risk model with cell chemotaxis, leukocyte migration, and regulation of leukocyte migration. Additionally, the ER stress-related risk model suggested the presence of an immunosuppressive microenvironment and immune checkpoint responses. We validated the significance of 7 ER stress-related genes obtained from LASSO regression analysis through RT-qPCR testing on osteosarcoma samples from a local hospital, and inferred the importance of STC2 based on the literature. Subsequently, IHC experiments using samples from 70 osteosarcoma cases and 21 adjacent tissue samples confirmed differential expression of STC2 between cancer and normal tissues, and explored the gene’s expression in pan-cancer and its association with clinical pathological parameters of osteosarcoma. In conclusion, we have proposed an ER stress risk model as an independent prognostic factor and identified STC2 as a novel risk indicator for disease progression, providing a promising direction for further research and treatment of osteosarcoma.

## 1 Background

Osteosarcoma (OS) is the most common primary bone tumor in children and adolescents (1, 2). OS is a malignant tumor with a high tendency for local invasion and metastasis (2). OS metastasis, especially to the lungs, is the most common cause of related deaths (3). Despite some progress made with multi-modal treatment (surgery, radiation therapy, chemotherapy, and immunotherapy), the survival rate of OS patients, particularly those with metastatic and recurrent disease, remains a major concern (4, 5). Meanwhile, pain management in osteosarcoma patients has garnered attention and has achieved some success (6, 7). Although many genes have been identified as potential biomarkers for predicting and treating OS (8), the heterogeneity and complexity of the OS genome seriously hinder progress in treatment. Therefore, there is an urgent need to better understand the mechanisms of OS tumor development and progression to identify more effective and specific biomarkers for early prediction, survival assessment, and treatment.

As the largest organelle in eukaryotic cells, the endoplasmic reticulum (ER), a membrane structure composed of branching tubules and flattened cisternae, is the main site for protein synthesis, processing, and transportation (9). However, the protein processing capacity of the ER is limited. When the protein folding capacity of the ER is exceeded, the cell is considered to be in an ER stress state (10). Many factors can affect the ER and lead to ER stress, thereby reducing the efficiency of protein folding, including oxidative stress, nutrient deprivation, protein toxicity, hypoxia and metabolic stress, and disrupted calcium balance (11, 12). It is generally believed that the three branches of transmembrane ER sensors, IRE1α, PERK, and ATF6, trigger ER stress. These three receptors continuously monitor misfolded proteins and when they reach a certain concentration, the sensors trigger the ER stress response (13). Some studies have confirmed that chronic ER stress is a typical feature of many diseases, including cancer (14).

In tumors, the high metabolism and proliferation of tumor cells lead to ischemia and hypoxia within the tumor, causing tumor cells to enter a state of sustained ER stress, which in turn affects tumor invasion and angiogenesis (15, 16). One branch of the ER, based on expression in cancer cells, can accelerate protein synthesis, folding, and secretion, increasing endoplasmic reticulum (ER) stress (15). Multiple studies have reported high levels of activation and expression of ER markers, including one major branch (IRE1α, PERK, and ATF6) and the related molecular chaperone GRP11, in many human cancers (lymphoma and myeloma) and solid tumors (breast, gastric, lung, liver, esophageal, and colon cancers) (17, 18). Research on ER stress (ERS) in cancer or tumors can help advance cancer or tumor therapy, and ERS can serve as a novel therapeutic target for cancer or tumors (9).

In this study, we developed an ER stress-related risk model that not only accurately predicts the prognosis of osteosarcoma patients but also distinguishes the immune features of osteosarcoma. Furthermore, we constructed a forest plot that combines the prognostic model with clinical pathological factors (age, gender, race, tumor metastasis, and primary tumor location) and found its performance in estimating the 1-year, 3-year, and 5-year survival rates of osteosarcoma patients to be outstanding.

We analyzed clinical samples of the 7 genes used to build the model and identified key genes that may serve as independent prognostic indicators. Additionally, we examined the clinical pathological significance of these key genes using public datasets and the use of a large number of tissue samples validated differences in expression between osteosarcoma and normal tissue. The study results highlight a key factor that integrates endoplasmic reticulum stress with tumor-specific expression differences, shedding light on a direction for further understanding the specific mechanisms and potential therapeutic approaches for osteosarcoma.

## 2 Method

### 2.1 Dataset and data collection

The mRNA microarray data was downloaded from the Gene Expression Omnibus (GEO) database (http://www.ncbi.nlm.nih.gov/geo/), with the accession number GSE14359. GeneCards (https://www.genecards.org/) is a searchable comprehensive database that provides user-friendly information about all annotated and predicted human genes. ER stress-related genes were extracted from GeneCards, and genes with a correlation score of ≥0 were selected. The TARGET database is derived from the XENA website, which compiles data from many databases and performs reasonable batch effect processing (https://xena.ucsc.edu/). Data preprocessing and differential gene expression (DEG) screening were performed using the limma R package from Bioconductor 3.8 (https://www.bioconductor.org/packages/release/bioc/html/limma.html) for quantile normalization of raw data and subsequent data processing to identify DEGs between osteosarcoma tissue and normal osteoblasts, as described above. The t-test was used to evaluate the DEGs between the two groups, and the false discovery rate (FDR) of the P-value was corrected using the Benjamini-Hochberg (BH) procedure. Only genes with a |log2 fold change (FC)| > 1 and FDR < 0.05 were selected. The PCA plot was used to analyze the reproducibility within sample groups and the differences between groups. Hierarchical clustering was used to analyze the differential gene expression patterns between the two sample groups. A volcano plot was used to visualize the significant DEGs after filtering.

### 2.2 Functional and pathway enrichment analysis of DEG

clusterProfiler V3.8 is an R package that relies on biological ontologies. It not only automatically performs the biological term classification process and gene cluster enrichment analysis, but also provides visualization modules for displaying the analysis results. In this study, the clusterProfiler package was used for gene ontology (GO) and Kyoto Encyclopedia of Genes and Genomes (KEGG) enrichment analysis of the identified DEGs.

### 2.3 Protein-protein interaction network

The STRING database (www.string-db.org) is an online database designed to identify PPI pairs and construct PPI networks from large protein functional groups. The STRING database was used to retrieve PPIs for the DEGs identified, and PPI pairs with a comprehensive score greater than 0.9 were selected. The Cytoscape software was then used to construct the PPI network of the DEGs, where each node represented a protein, and the number of edges corresponded to the degree of interaction. CytoNCA V2.16 is a Cytoscape plugin used for network centrality analysis, which can be used to identify key nodes (genes) in the network. In this study, four typical centrality indices were used to identify key genes: eigenvector centrality, degree centrality, betweenness centrality, and closeness centrality. Finally, the top 12 genes in the PPI network were identified as key genes based on their centrality values.

### 2.4 Construction and validation of ER pressure-related risk characteristics

We first obtained the intersection of differentially expressed genes (DEGs) and ER stress-related genes to obtain a set of differentially expressed ER stress-related genes (DERs). Using the survival R package, we performed univariate Cox regression and Kaplan-Meier (KM) analysis to identify DERs associated with overall survival (OS) time of patients, including the TGRGET dataset. Only genes with p-values ≤ 0.05 in both analyses were included in the next step.

Using the glmnet R package in the TGRGET database, we performed LASSO regression analysis on the cross genes related to OS in the above dataset to narrow down the range of prognostic-related genes. Then, we used the Akaike information criterion (AIC) method in the survival package to perform multivariate Cox regression analysis. Based on the linear integration of the regression coefficients obtained from multivariate Cox regression analysis and the expression levels of selected DERs, we established the optimal ER stress-related risk feature.

The experience here refers to the expression values of ER stress-related genes and the corresponding regression coefficients calculated through multivariate Cox regression analysis. The TARGET database was randomly divided into two databases, with TARGET-A used as the training set and TARGET-B used as the validation set.

### 2.5 Survival analysis

The Kaplan-Meier survival analysis was conducted using the survival and survminer packages in R to compare the overall survival (OS) among different groups of glioma patients. The survivalROC package in R was used to establish time-dependent receiver operating characteristic (ROC) curves to examine the accuracy of risk features in predicting the outcome of glioma patients. The larger the area under the ROC curve (AUC), the stronger the predictive ability of the risk model. The Pheatmap package in R was used to create a risk map, displaying the distribution of survival status among different risk groups of samples.

### 2.6 Functional enrichment analysis

The GSVA package in R was utilized to estimate gene ontology (GO) biological processes and Kyoto Encyclopedia of Genes and Genomes (KEGG) pathways associated with risk features. The GSVA package scores the GO biological processes and KEGG pathways in each sample, and by comparing the score differences among different risk groups, we identified different biological processes enriched in the high-risk and low-risk groups. The limma package in R was employed to identify differentially expressed genes and gene sets in different populations. To further validate the GO processes and KEGG pathways associated with the signature, the clusterProfiler package in R was used to perform GO and KEGG analyses on differentially expressed genes. All heatmaps were created using the Pheatmap package in R.

### 2.7 Independent prognostic effects of risk features and development of a histogram

To determine if ER stress-related risk features are dependent on other clinical pathological factors (including age, gender, race, tumor metastasis, and tumor primary site) for predicting the OS of patients, univariate and multivariate Cox regression analyses were performed using the survival package in R. The results of the independent prognostic factor analysis were displayed in the form of a forest plot using the forest package in R. Column line charts were used to create personalized prediction models by visually displaying the probability of clinical events based on the predicted model. Age, gender, race, tumor metastasis, tumor primary site, and ER stress-related risk score were combined, and column line charts were developed using the survival and rms packages in R.

### 2.8 Transcriptional quantitative polymerase chain reaction

We obtained three osteosarcoma specimens (n=3) from the Department of Spine and Bone Oncology of Wuhan University and sent them to Wuhan Sevier Biotechnology Co., LTD., for RNA extraction. The action of endogenous genomic DNA was removed by a specific DNA enzyme (Thermo Scientific, K2981) and then cDNA was synthesized by RevertAid RT reverse transcription kit (Thermo Scientific, K1691). Finally, mRNA was quantified by quantitative real-time PCR using SYBR Color qPCR mixture (Vazyme, Jiangsu, China) and LightCycler480 real-time fluorescent quantitative PCR system. The PCR primer sequence is shown in **Table 1**. Comparative calculation of relative mRNA−ΔΔCt method was used.

**Table 1:** Primers for quantitative RT-PCR.

Gene	Forward Primer Sequence (5′-3′)	Reverse Primer Sequence (5′-3′)
**ACTB**	AGATCAAGATCATTGCTCCTCCT	ACGCACCTCAGTAACAGTCC
**STC2**	CCTGCAGAATACAGCGGAGA	GCCCCGAATCTCACAAGAGT
**TNFRSF11B**	AGACGTCATCTAAAGCACCCTG	TCCTCACACAGGGTAACATCTATTC
**SCD5**	CTTGGCCTCTATTCTCCGCTA	ATTATGGAAGCCTTCACCAATGGC
**GRN**	AGTCGGACGCAGGCAGA	GTTGTGGGCCATTTGTCCAG
**PLCB1**	GGTGCAGTATATCAAGAGGCTAGA	CTGCAGCTTGGGCTTTTCAT
**GLB1**	TTGCGCAATGCCACCCA	CAGGGCACATACGTCTGGAT
**CRAT**	CCGAAGGCTCTAGCAAGGAC	CAGAGGCTTCACCACGGTC

### 2.9 Immunohistochemistry

We obtained pathological slides of 70 cases of osteosarcoma and 21 cases of adjacent cancer tissues, purchased from Zhongke Guanghua (Xi’an) Intelligent Biotechnology Co., Ltd. The slides were baked at 60°C for 30 minutes and then underwent routine deparaffinization and hydration. Antigen retrieval buffer (pH 6.0 citrate buffer) was added to a pressure cooker for retrieval, heated at medium heat for 10 minutes until boiling, followed by a 5-minute cooling period, and then transferred to medium-low heat for another 5 minutes for antigen retrieval. After cooling to room temperature, the slides were washed with phosphate-buffered saline (PBS) for 5 minutes × 3 times. Endogenous peroxidase was blocked with 3% H202-methanol at room temperature for 30 minutes, followed by a PBS wash for 5 minutes × 3 times. Normal non-immune animal serum was added and incubated at room temperature for 10 minutes; the serum was then removed and primary antibody Anti-STC2 (proteintech, 10314-1-AP, 1:300) was added and incubated overnight at 4°C. The slides were washed with 0.1% Tween-20 PBS for 5 minutes × 3 times, followed by the addition of enzyme-labeled polymer secondary antibody and incubation in a humid chamber at 37°C for 30 minutes. After washing with 0.1% Tween-20 PBS for 5 minutes × 3 times, DAB staining was performed for 5 minutes, and the reaction was stopped with distilled water. Counterstaining was carried out with hematoxylin, followed by rinsing, differentiation, and thorough rinsing with running tap water. The slides were then dehydrated, cleared, and mounted with neutral gum.

The evaluation criteria for the IHC experiment are as follows: After locating the staining results on the chip point by point, the cell staining intensity is determined as follows: no staining is considered negative (-), light brown staining is considered weak positive (+), brown staining is considered positive (++), and dark brown staining is considered strong positive (+++). The number of positive cells is determined as the ratio of target cells expressing the target protein to all target cells.

### 2.10 Big data analysis of osteosarcoma: the relationship between STC2 and osteosarcoma

The big data of osteosarcoma is sourced from research institutions in 86 countries and regions worldwide. It includes detailed clinical information data of 495 osteosarcoma patients from three osteosarcoma research projects. The osteosarcoma tissues of these patients have undergone high-throughput detection of genomics, transcriptomics, proteomics, metabolomics, and microbiomics, resulting in a comprehensive, high-quality dataset that provides detailed insights into the molecular biology and clinical practice of osteosarcoma. This dataset is of great importance as a research resource for osteosarcoma.

### 2.11 Statistical analysis

R software (version 3.6.3) and GraphPad Prism v7.00 (GraphPad Software Inc.) were used as statistical analysis tools for this study. Quantitative data are presented as mean ± standard error of the mean (SEM) or standard deviation (SD). The Wilcoxon test was used to compare statistical differences between two groups, and the Kruskal-Wallis H test was used to compare multiple groups. Statistical significance was defined as P <.05. Other plots were constructed using R software or GraphPad Prism. The Fisher’s exact test and chi-square test were used to analyze the correlation between STC2 protein expression intensity and categorical clinical factors.

## 3 Results

### 3.1 Identification of DEG

**Figure 1:**
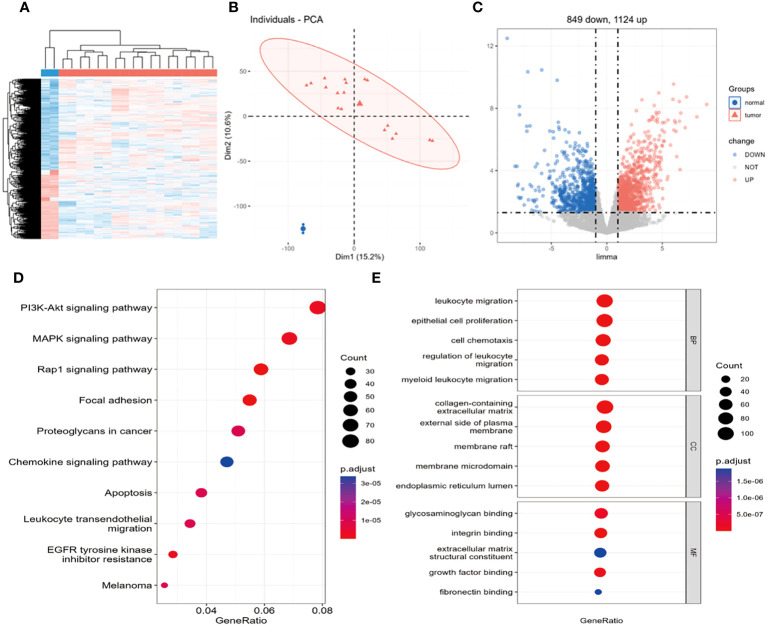
Identification of 1973 differentially expressed genes and KEGG, GO enrichment analysis. **(A)** Heatmap of differentially expressed genes, **(B)** PCA plot, **(C)** volcano plot. **(D)** Bubble plot of KEGG enrichment analysis signaling pathways, **(E)** Bubble plot of GO enrichment analysis biological processes. In **(A)**, the blue group represents non-tumor primary osteoblasts, the red group represents osteosarcoma, with red indicating high expression and blue indicating low expression. In **(B)**, the grouping is the same as in **(A)**; this plot shows strong repeatability within the normal and tumor groups, with large differences between groups. In **(C)**, black dots represent genes with no differential expression, red dots represent upregulated DEGs, and blue dots represent downregulated DEGs.

**Figure 2:**
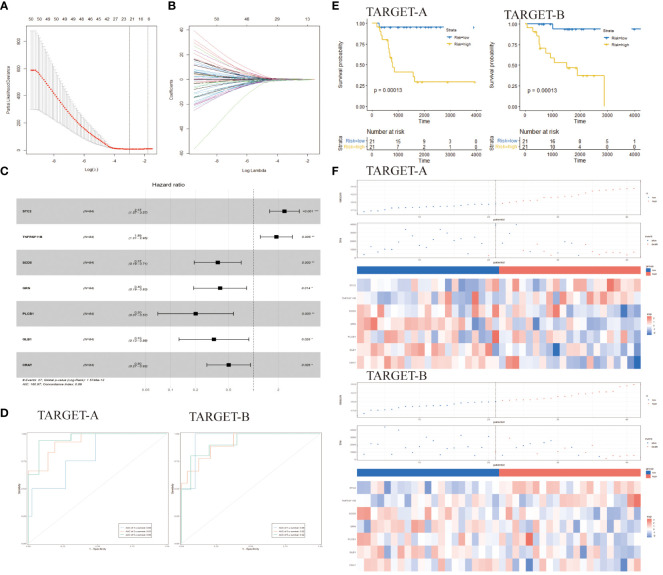
Identification of prognostic genes for developing a risk model and assessing the prognostic predictive ability of endoplasmic reticulum stress-related risk features. **(A)** LASSO coefficient spectrum of 59 genes in the TARGET-A dataset. **(B)** Selection of the optimal parameter (λ) in the LASSO model. **(C)** Selection of <7> genes to determine prognostic features. **(D)** ROC curves of 7 gene markers in the TARGET-A and TARGET-B cohorts. **(E)** KM curves of prognostic features in the TARGET-A and TARGET-B cohorts **(C, D)** (log-rank test). **(F)** Risk score distribution in the TARGET-A and TARGET-B cohorts. Chinese glioma genome map; ER, endoplasmic reticulum; LASSO, Least Absolute Shrinkage and Selection Operator; ROC, Receiver Operating Characteristic; Cancer genome atlas LASSO, Least Absolute Shrinkage and Selection Operator; OS, Overall Survival; TARGET, The Cancer Genome Atlas.

After data normalization, differential gene expression analysis was performed using a dataset containing 18 osteosarcoma samples and 2 non-tumor primary osteoblast control samples. The heatmap shows a large number of differentially expressed genes, with a clear distribution between groups (**Figure 1A**). The PCA plot demonstrates significant differences between sample groups and good repeatability within groups (**Figure 2B**). Based on the criteria of logFC=1 and P.Value=0.05, a total of 22,283 DEGs were identified. Among these genes, 1,124 were upregulated, and 849 were downregulated (**Figure 1C**).

### 3.2 Enrichment analysis of KEGG and GO pathways

To further investigate the potential functions of DEGs at a more practical level, the latest versions of the GO and KEGG pathway databases were utilized to analyze genes and determine the potential functions of DEGs. The KEGG results revealed enrichment mainly in the PI3K/AKT pathway, MAPL pathway, Rap1 pathway, and focal adhesion pathway related to cell migration (**Figure 1D**). These pathways have a certain impact on the occurrence and development of osteosarcoma (OS), with the PI3K signaling pathway potentially playing a crucial role in OS development. Previous studies have reported the significant role of the PI3K signaling pathway in cell proliferation (23, 24), which is vital for the development of OS. The GO enrichment analysis indicated significant enrichment of DEGs in 1294 biological processes (BPs), 85 molecular functions (MFs), and 100 cellular components (CCs). The GO results mainly involve cell chemotaxis, leukocyte migration, and regulation of leukocyte migration (**Figure 1E**).

### 3.3 Identification of differential genes and ER stress-related genes to establish a risk correlation model

A total of 7,070 ER stress-related genes were extracted from the GeneCards database with a relevance score of ≥0. By intersecting these genes with the 1,973 differential genes obtained earlier, a DERs gene set consisting of 963 genes was generated. Univariate Cox regression and KM analysis were conducted on these genes. The TARGET database was randomly divided into two subsets, namely TARGET-A and TARGET-B. In the TARGET-A dataset, 59 genes were found to be significantly associated with osteosarcoma (OS) patients (**Figure 2A**). The overlapping genes (59 genes) were included in the LASSO regression analysis to prevent overfitting issues in risk features (**Figure 2B**). The AIC method of multivariate Cox regression analysis was applied to the genes returned by the LASSO regression analysis (7 genes) to construct the optimal model, which included STC2, TNFRSF11B, SCD5, GRN, PLCB1, GLB1, and CRAT (**Figure 2C**).

### 3.4 Establish and evaluate risk characteristics related to ER stress

The cancer genome atlas osteosarcoma data was used to construct risk features. Time-dependent ROC curves were utilized to evaluate the efficiency of prognostic prediction for ER stress-related risk features. In the TARGET-A dataset, the AUC for 1-year, 3-year, and 5-year overall survival (OS) were 0.80, 0.93, and 0.96 respectively; while in the TARGET-B dataset, the AUC for predicting 1-year, 3-year, and 5-year OS were 0.95, 0.92, and 0.92 (**Figure 2D**). Samples in TARGET-A and TARGET-B were stratified into low-risk and high-risk groups based on the median risk score of each cohort. KM analysis revealed that patients in the low-risk group had better outcomes than those in the high-risk group (**Figure 2E**). In the TARGET-A dataset, the three-year OS rates for the high-risk and low-risk groups were 40.44% vs 86.55%, while in the TARGET-B cohort, they were 52.77% vs 93.75%. **Figure 2F** depicts a traditional tripartite linkage illustrating the comparison of survival and mortality outcomes after stratifying into high and low-risk groups based on risk scores.

### 3.5 A high ER stress-related risk score showed immunosuppressive characteristics

**Figure 3:**
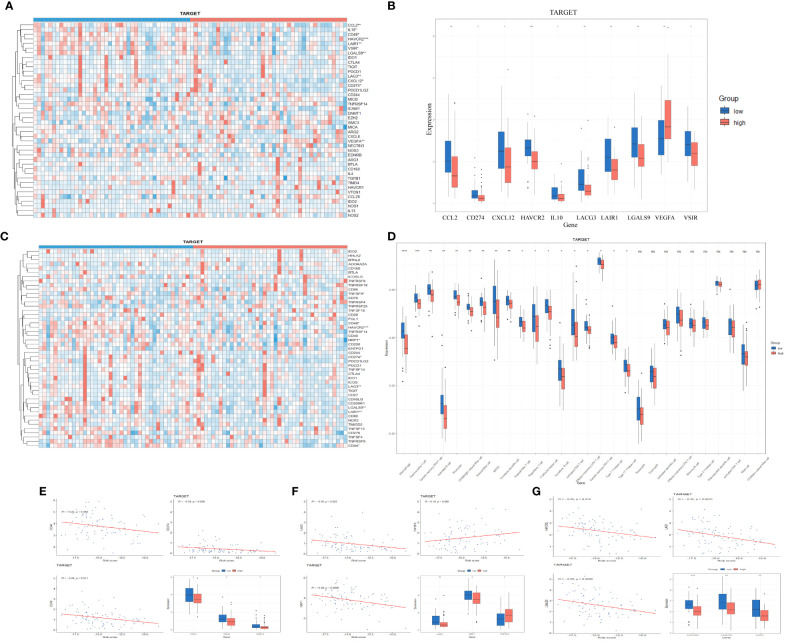
**(A, B)** Expression of negative regulators of cancer-immunity cycle in low-risk and high-risk groups in TARGET database (Wilcoxon test). **(C, D)** Expression of immune checkpoints in low-risk and high-risk groups in TARGET database (Wilcoxon test). **(E–G)** Correlation between endoplasmic reticulum stress risk score and expression of multiple important genes in TARGET cohort (Pearson correlation analysis).

The process of immune system eradication of tumors involves the cancer-immunity cycle. We explored the expression characteristics of genes that promote this cycle in the TARGET dataset. These genes were obtained from the Tumor Immune Phenotype (TIP) website (http://biocc.hrbmu.edu.cn/TIP/index.jsp). As shown in **Figures 3A, B**, most of these genes are downregulated in the high-risk group, inhibiting the cycle of immune system eradication of tumors. The immune checkpoint ligand CD274 (programmed death-ligand 1, B7-H1) inhibits anti-tumor immunity by interacting with the PDCD1 (programmed cell death 1, PD-1) receptor on T lymphocytes in various tumors (19, 20).

In addition to the above-mentioned inhibitors of the cancer-immunity cycle, immune checkpoints can also suppress the ability of the immune system to clear tumors. In recent years, immune checkpoints have emerged as potential therapeutic targets in many malignant tumors and play a crucial role in tumor immunotherapy. A comparison of immune checkpoint expression between the high-risk and low-risk groups in the TARGET cohort is shown in **Figures 3C, D**. Considering the critical role of TNFSF9 in tumor immune suppression and immunotherapy, we studied the relationship between its expression levels and ER stress-related risk scores. We found a significant positive correlation between TNFSF9 expression levels and risk scores (**Figure 3F**), with higher expression levels observed in the high-risk group compared to the low-risk group (**Figure 3D**). We also examined the expression levels of some conventional immune checkpoint genes, such as CD44 (21), CD48 (22), CD274 (19), LAG3 (23), NRP1 (24), HAVCR2 (25), LGALS9 (26), LAIR1 (27), which showed a significant negative correlation with risk scores (**Figures 3E–G**). These data suggest that ER stress-related risk features can accurately predict the immune characteristics of gliomas.

### 3.6 Construction of line diagrams and feature verification

**Figure 4:**
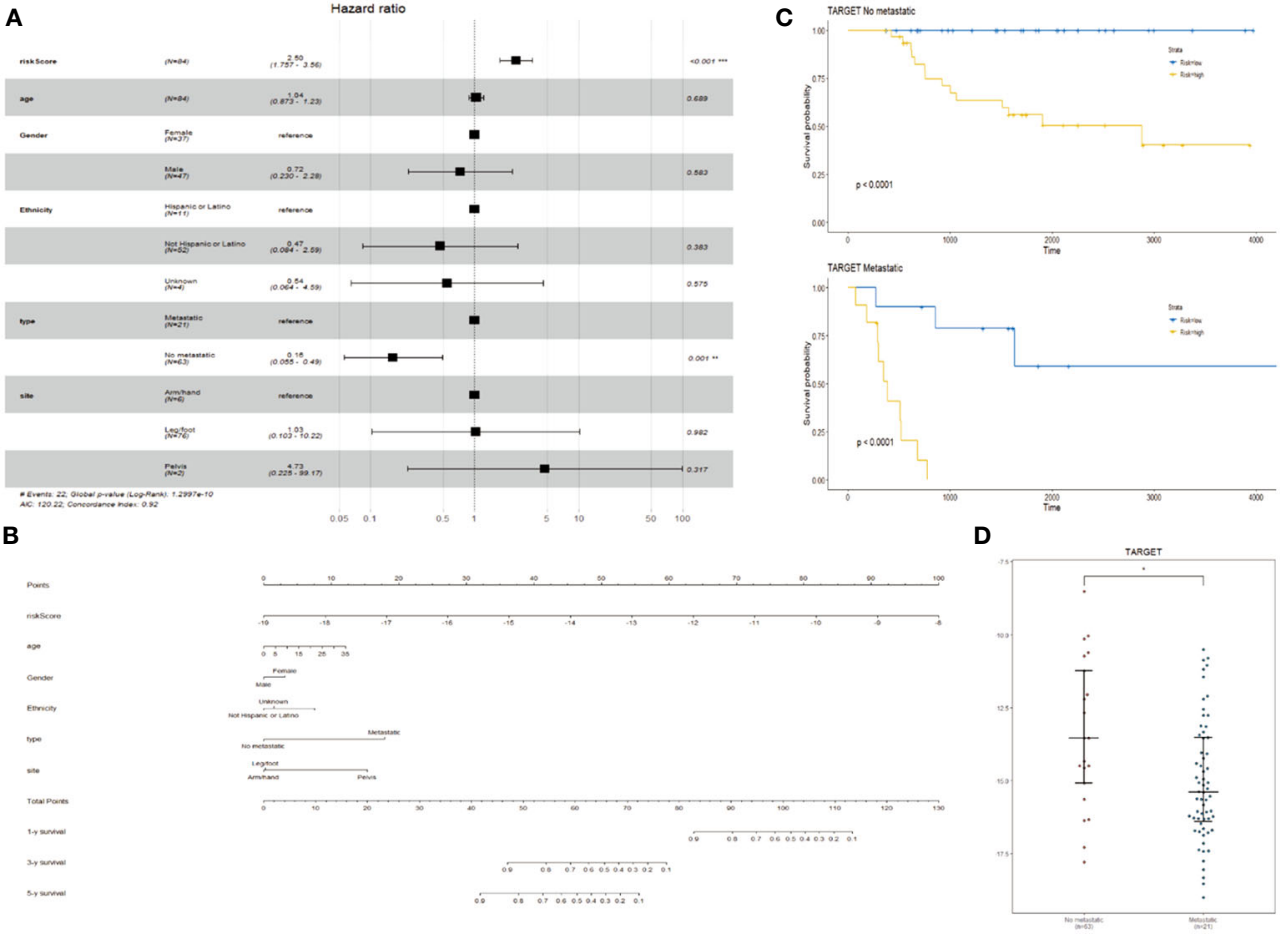
Creation of column charts. **(A)** Forest plot of multivariate Cox regression analysis in TARGET cohort. **(B)** Column chart constructed based on stress risk features, age, sex, race, metastasis, and primary site. **(C, D)** KM curves **(C)** (log-rank test) and beeswarm plots **(D)** of prognostic features in TARGET cohort for tumors with and without metastasis.

In addition to ER stress risk scores, there are many known prognostic factors for osteosarcoma, such as age, gender, race, metastasis status, and primary site. Therefore, it is necessary to examine whether ER stress risk features can independently predict prognosis. In the training cohort of TARGET, univariate Cox analysis shows a positive correlation between ER stress-related risk scores and the overall survival (OS) of osteosarcoma patients. Furthermore, age, gender, race, metastasis status, and primary site are also significantly associated with OS (**Figure 4A**). Subsequent multivariate Cox regression analysis indicates a significant association between ER stress-related risk features, metastasis status, and OS (**Figure 4B**). These findings suggest that the ER stress-related risk features constructed using the TARGET dataset are independent prognostic factors for osteosarcoma patients.

By integrating ER stress risk features, age, gender, race, metastasis status, and primary site, we constructed a nomogram to predict 1-year, 3-year, and 5-year OS in the TARGET dataset. In the nomogram, each signature is assigned a score based on its contribution to the risk of OS (**Figure 4B**). Additionally, metastasis status is considered as an independent risk factor, and survival and expression analyses are conducted (**Figures 4C, D**).

### 3.7 Differential expression of STC2 in osteosarcoma

**Figure 5:**
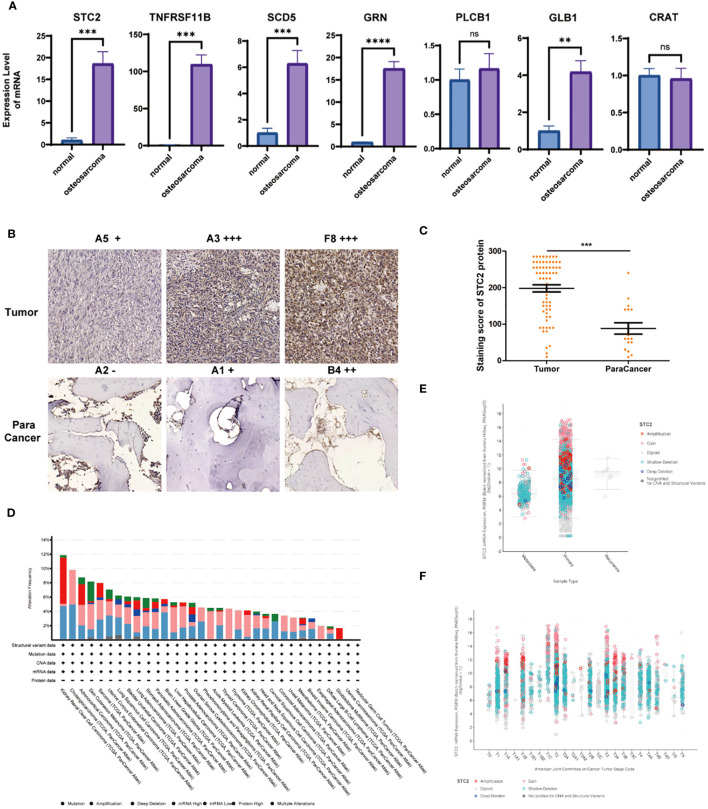
Analysis of STC2 in osteosarcoma. **(A)** Comparison of key gene RNA expression levels obtained from osteosarcoma and normal tissues using RT-qPCR. **(B)** IHC staining of STC2 in clinical samples of osteosarcoma and adjacent non-cancerous tissues. **(C)** Quantitative analysis of IHC staining (*** indicates Wilcoxon test P < 0). **(D)** Analysis of the variation statistics of STC2 in multiple cancer types. **(E)** Relationship between STC2 gene expression and TNM Stage. **(F)** Association with primary, metastatic, and recurrent cases.

Our forest plot revealed 7 genes closely associated with patient survival. To further validate the correlation between these genes and osteosarcoma, we obtained the RNA expression of these 7 genes from samples of osteosarcoma patients through RT-qPCR. We found that genes with a hazard ratio >1 (STC2 and TNFRSF11B) were expressed at higher levels in tumor tissues compared to normal tissues, with a significant and meaningful increase (**Figure 5A**). There was no difference in the expression of PLCB1 and CRAT between tumor and normal tissues. Therefore, we speculate that STC2 and TNFRSF11B may play a more critical role in the progression of osteosarcoma. STC2, a glycoprotein, is expressed in a wide range of tumor cells and tissues such as human breast, colon, stomach, esophagus, prostate, kidney, liver, bone, ovary, and lung, with its overexpression promoting cell proliferation, migration, and immune response.

Subsequently, using a large number of clinical samples, we conducted IHC staining and found that STC2 expression in tumor tissues was significantly higher than in adjacent non-cancerous tissues (**Figure 5B**). Through pathologist’s metastasis analysis and statistics, we confirmed the high expression of STC2 in tumor tissues (**Figure 5C**). By analyzing cancer databases, we examined the variation statistics of STC2 in multiple cancer types (**Figure 5D**), the gene expression of STC2 in relation to TNM Stage (**Figure 5E**), and its association with primary, metastatic, and recurrent cases (**Figure 5F**).

## 4 Discussion

A large body of evidence suggests that a certain level of endoplasmic reticulum (ER) stress promotes cancer progression through mechanisms such as cell proliferation, metastasis, vascular resistance, and treatment resistance (15, 16). The tumor microenvironment emphasizes the disruption of protein homeostasis that can generate ER stress, which can be counteracted by triggering the unfolded protein response (UPR) (28). At the same time, a certain degree of ER stress can activate cell protective mechanisms, thereby promoting the cell’s immune response (29). Furthermore, cancer cells under ER stress release unknown factors that induce ER stress in macrophages, leading to the release of pro-inflammatory cytokines. In various cancers such as lymphoma, neuroblastoma, prostate cancer, and breast cancer, excessive ER stress can induce carcinogenic transformation, leading to the overactivation of MYC in normal epithelial cells, causing protein toxicity stress, reducing cell survival, and weakening the competitive ability of cancer cells (30). Overall, these findings suggest that investigating the mechanisms of ER stress in cancer development and its relationship with the immune microenvironment may contribute to the prediction of cancer and ER stress-related immunotherapy.

In osteosarcoma, ER stress has significant implications for tumor occurrence, development, and treatment (31–33). However, the role of ER stress in the anti-tumor immune response in osteosarcoma is not yet clear. Based on the above content, we speculate that ER stress may affect the immune features of the osteosarcoma microenvironment.

In this study, we downloaded samples of osteosarcoma and osteoblasts from the Gene Expression Omnibus (GEO) database (http://www.ncbi.nlm.nih.gov/geo/) under accession number GSE14359 and performed differential analysis using the limma R package, resulting in 1973 differentially expressed genes. We then performed KEGG and GO enrichment analysis and PPI protein network construction. We retrieved and downloaded 7070 ER stress-related genes from the GeneCards website and intersected them with the 1973 differentially expressed genes to obtain a set of 963 DERs genes. Further LASSO regression analysis and multivariate Cox regression analysis identified 7 OS-related genes (STC2, TNFRSF11B, SCD5, GRN, PLCB1, GLB1, CRAT), and an OS-related prediction model was constructed. The expression levels of STC2 and TNFRSF11B were positively correlated with a good outcome, while the expression levels of SCD5, GRN, PLCB1, GLB1, and CRAT were negatively correlated with a good outcome.

In addition, among the 7 OS-related genes, STC2 (34) and TNFRSF11B (35, 36) are upregulated to promote cancer progression, while SCD5 (36) and CRAT (37) inhibit cancer development through certain mechanisms. STC2 is a glycoprotein expressed in a broad range of tumor cells and tissues, such as human breast, colon, stomach, esophagus, prostate, kidney, liver, bone, ovary, and lung, and its overexpression promotes cell proliferation, migration, and immune response (34). TNFRSF11B is highly expressed in colon cancer and inhibits the infiltration of memory CD4+ T cells in the colon cancer microenvironment, thereby weakening the immune killing response to cancer cells (35). Moreover, TNFRSF11B can also activate Wnt/β-catenin signaling and promote gastric cancer progression (36). SCD5 is an integral membrane protein involved in lipid metabolism in the endoplasmic reticulum, and its presence can optimize the prognosis of breast cancer and improve the responsiveness to neoadjuvant chemotherapy (36). The agonist of CRAT can promote the metastasis of melanoma (37). Therefore, it is not surprising that most of the identified 7 genes directly or indirectly affect the function of immune cells, indicating that ER stress may also regulate the immune response to osteosarcoma. Furthermore, circular RNAs and microRNAs are closely associated with the prognosis of osteosarcoma. This study focuses on investigating target genes, and exploring how these genes are affected can be further explored through non-coding RNAs, providing deeper insights (38).

Risk scoring is a commonly used method for developing meaningful signatures. The model developed using the ER stress-related risk scoring not only accurately predicts the prognosis of osteosarcoma patients but also distinguishes different molecular subtypes of gliomas. ROC analysis showed that the gene markers performed well in predicting the short-term (3 and 5 years) and long-term (10 years) survival of glioma patients in the TARGET-A and TARGET-B datasets. KM analysis confirmed that the model accurately predicts the survival rate of osteosarcoma patients.

Considering the strong impact of these risk features in gliomas, we further evaluated the mechanisms of these effects. Functional analysis showed enrichment of biological processes related to cell chemotaxis, leukocyte migration, and regulation of leukocyte migration in the high-risk group, indicating an interaction between ER stress and the immune response in gliomas. Compared to the low-risk group, the high-risk group exhibited high expression of cancer immune cycle inhibitors and immune checkpoint molecules, as well as enrichment of tumor immune suppressive cells, demonstrating successful classification of the immune type of osteosarcoma by this model. This suggests that ER stress can regulate the immune microenvironment of osteosarcoma, affecting the prognosis of glioma patients, and confirms our hypothesis regarding the relationship between ER stress and anti-glioma immune response is correct.

To fully leverage the potential of the risk model, we developed a forest plot that combines ER stress risk features, age, gender, race, metastasis status, and primary site. Calibration plots and DCA based on the TARGET database demonstrated the excellent predictive performance of the forest plot. Therefore, our 7 genes associated with ER stress risk features can predict the overall survival of osteosarcoma patients and facilitate the selection of optimal treatment methods. We selected the most representative gene, STC2, from the 7 genes using RT-qPCR with samples from local hospitals. Subsequently, through IHC staining with a large number of samples, we analyzed the high expression of STC2 in osteosarcoma patients. Finally, we analyzed the relationship between STC2 and pan-cancer as well as various clinical factors using large databases.

However, our study also has some limitations. Due to the large size of osteosarcoma tumor tissues, we can only validate a portion belonging to the tumor tissue during experimental verification. Subsequent experiments can improve accuracy by sampling multiple regions of the larger tissue in different zones. Further research is needed to confirm the specific functional mechanisms of ER stress-related risk features in osteosarcoma. Endoplasmic reticulum stress of osteosarcoma cells can be activated by exogenous stimulation to determine the downstream phenotype. Despite its outstanding performance in distinguishing osteosarcoma survival differences and immune characteristics, the accuracy of the risk model in discriminating between normal bone tissue and osteosarcoma tissue requires further investigation. The mouse and rat osteosarcoma models can be used for multi-species validation.

In conclusion, our study provides important resources for elucidating the specific role of ER stress in osteosarcoma and reveals STC2 as an important risk indicator for disease progression, offering new insights for mechanistic research and treatment of osteosarcoma from the perspective of endoplasmic reticulum stress.

## 5 Conclusion

We combined the differentially expressed genes identified through analysis of the GEO database with endoplasmic reticulum stress-related genes to construct an osteosarcoma ER stress prognostic model using the TARGET database as the training set. We also validated the critical significance of the endoplasmic reticulum stress-related gene STC2 in osteosarcoma, providing promising insights for the mechanistic understanding and treatment of osteosarcoma.

## Data availability statement

The original contributions presented in the study are included in the article/supplementary material. Further inquiries can be directed to the corresponding author.

## Ethics statement

The studies involving humans were approved by the Medical Ethics Committee of Zhongnan Hospital of Wuhan University. The studies were conducted in accordance with the local legislation and institutional requirements. The participants provided their written informed consent to participate in this study.

## Author contributions

YY: Conceptualization, Methodology, Supervision, Writing – original draft, Writing – review & editing. JY: Funding acquisition, Investigation, Methodology, Project administration, Writing – original draft, Writing – review & editing. ZP: Writing – original draft, Writing – review & editing.
